# Engineering CGTase to improve synthesis of alkyl glycosides

**DOI:** 10.1093/glycob/cwaa109

**Published:** 2020-12-02

**Authors:** Kazi Zubaida Gulshan Ara, Javier A Linares-Pastén, Jonas Jönsson, Maria Viloria-Cols, Stefan Ulvenlund, Patrick Adlercreutz, Eva Nordberg Karlsson

**Affiliations:** Biotechnology, Department of Chemistry, Lund University, P.O. Box 124, 22100 Lund, Sweden; Biotechnology, Department of Chemistry, Lund University, P.O. Box 124, 22100 Lund, Sweden; Biotechnology, Department of Chemistry, Lund University, P.O. Box 124, 22100 Lund, Sweden; Biotechnology, Department of Chemistry, Lund University, P.O. Box 124, 22100 Lund, Sweden; Enza Biotech AB, Scheelevägen 22, 22363 Lund, Sweden; Enza Biotech AB, Scheelevägen 22, 22363 Lund, Sweden; Biotechnology, Department of Chemistry, Lund University, P.O. Box 124, 22100 Lund, Sweden; Biotechnology, Department of Chemistry, Lund University, P.O. Box 124, 22100 Lund, Sweden

**Keywords:** acceptor subsites, cyclodextrin glycosyltransferase, coupling reaction, dodecyl-β-maltoside, γ-Cyclodextrin

## Abstract

Alkyl glycoside surfactants with elongated carbohydrate chains are useful in different applications due to their improved biocompatibility. Cyclodextrin glucanotransferases can catalyze the elongation process through the coupling reaction. However, due to the presence of a hydrophobic tail, the interaction between an alkyl glycoside acceptor and the active site residues is weaker than the interaction with maltooligosaccharides at the corresponding site. Here we report the mutations of F197, G263 and E266 near the acceptor subsites in the CGTase *C*spCGT13 from *Carboxydocella* sp. The results showed that substitutions of both F197 and G263 were important for the binding of acceptor substrate dodecyl maltoside during coupling reaction. The double mutant F197Y/G263A showed enhanced coupling activity and displayed a 2-fold increase of the primary coupling product using γ-cyclodextrin as donor when compared to wildtype *C*spCGT13. Disproportionation activity was also reduced, which was also the case for another double mutant (F197Y/E266A) that however not showed the corresponding increase in coupling. A triple mutant F197Y/G263A/E266A maintained the increase in primary coupling product (1.8-fold increase) using dodecyl maltoside as acceptor, but disproportionation was approximately at the same level as in the double mutants. In addition, hydrolysis of starch was slightly increased by the F197Y and G263A substitutions, indicating that interactions at both positions influenced the selectivity between glycosyl and alkyl moieties.

## Introduction

Cyclodextrin glucanotransferases (CGTase; E.C 2.4.1.19) belonging to the glycoside hydrolase family 13 (GH 13) are widely used as catalysts in starch conversion processes (Han et al. 2014). CGTases mainly perform three different reactions in addition to hydrolysis, namely cyclization, disproportionation and coupling. The cyclization reaction takes place via intramolecular transglycosylation to produce cyclodextrin, which is unique to CGTases (Qi and Zimmermann 2005). On the other hand, both disproportionation and coupling reactions involve transglycosylation where malto-oligosaccharides are transferred to different acceptor molecules resulting in glycosylated conjugates (van der Veen et al. 2000). During this intermolecular transglycosylation, the coupling reaction involves the ternary complex mechanism (Nakamura et al. 1994) where both acceptor and donor substrate can bind simultaneously. In the disproportionation reaction, it is a substituted-enzyme mechanism (van der Veen et al. 2000) where the donor (linear maltooligosaccharide) is cleaved and transferred to an acceptor (also a linear maltooligosaccharide) but during the transfer process it also generates a by-product (leaving group) which leaves the glycosyl-intermediate followed by binding of the acceptor substrate. Coupling and disproportionation reactions have been used to generate a wide range glycosylated derivatives to obtain new products, such as 2-O-α-D-glucopyranosyl-L-ascorbic acid (AA-2G), and different modified alkyl glycosides (Svensson et al. 2009b; Leemhuis et al. 2010; Chotipanang et al. 2011; Han et al. 2012).

Alkyl glycosides are nonionic surfactants that can be produced from renewable resources, using fatty alcohols and plant-based polysaccharides like starch (Vemula and John 2008; Hayes and Smith 2019). Alkyl glycosides have gained huge attention because of their various advantages including biodegradability and low toxicity. These properties make them desirable for numerous applications in the detergents and cosmetics areas. In addition to that, they are also used for the extraction and crystallization of membrane proteins or for transfection (von Rybinski and Hill 1998; Blunk et al. 2006). In most cases, commercially available alkyl glycosides are generated by the acid catalyzed Fischer process containing on average 1–2 D-glucosyl units (Fischer and Helferich 1911). Alkyl glycosides can, however, also be obtained via enzyme catalyzed synthesis, in transglycosylation reactions of retaining glycoside hydrolases. In this field, exo-acting glycosidases have predominantly been used, limiting transfer to monosaccharide moieties, resulting in products with single monosaccharide head-groups (Ljunger et al. 1994; Hansson and Adlercreutz 2002; Rather et al. 2012; Lundemo et al. 2013). However, alkyl glycosides with a higher degree of polymerization (DP) of the carbohydrate moiety is more required since it has been shown for other nonionic surfactants that elongation of the hydrophilic head-group makes the surfactant less toxic to cells and tissue (Ekelund et al. 2005). The elongation of the carbohydrate head-group can be achieved by CGTases through the coupling reaction between α-cyclodextrin and alkyl glycosides resulting in longer chain length of the head-group. However, during this process disproportionation and hydrolysis reactions can also occur to a certain extent (Svensson et al. 2009a, 2009b).

In our previous studies we have characterized a novel CGTase (*C*spCGT13) from the genus *Carboxydocella*. The coupling activity of *C*spCGT13 was also compared with other CGTases and it showed high coupling activity between gamma cyclodextrin (γ-CD) and an alkyl glycoside, with a dodecyl alkyl chain (Ara et al. 2015). However, in a later study *C*spCGT13 demonstrated higher coupling activity with methyl-α-glucopyranoside as acceptor (Rather et al. 2015). The increased hydrophobicity of the alkyl glycoside thus weakens its capacity to act as acceptor molecule. Despite its high coupling activity, *C*spCGT13 also has significant hydrolytic activity, which limits the usability of this enzyme for producing alkyl glycosides with defined length of the carbohydrate chain. Hence, it is important to investigate the influence of acceptor and donor subsite residues in determining the specificity profile of the enzyme. There have been several mutational studies done for CGTases to investigate their product specificity profiles for conventional substrates, like starch and maltooligosaccharides. Nonetheless, the coupling activity has not been the main focus of any studies, and the interactions between alkyl glycosides and the sub-sites have not been studied before.

In the present study *C*spCGT13 was engineered via site-directed mutagenesis. The focus of this mutational study was the acceptor subsites, in order to achieve more favorable interactions with the alkyl glycoside. Also, it was previously shown that the hydrophobic residues in the acceptor subsite can play a significant role in determining the reaction specificity (van der Veen et al. 2001). Here we report the characterization of seven mutants and the wildtype *C*spCGT13 based on their coupling and disproportionation activity, using an alkyl glycoside as acceptor and γ-cyclodextrin as the donor.

## Results and discussions

### Structural analysis and residue selection for site-directed mutagenesis

**Fig. 1:**
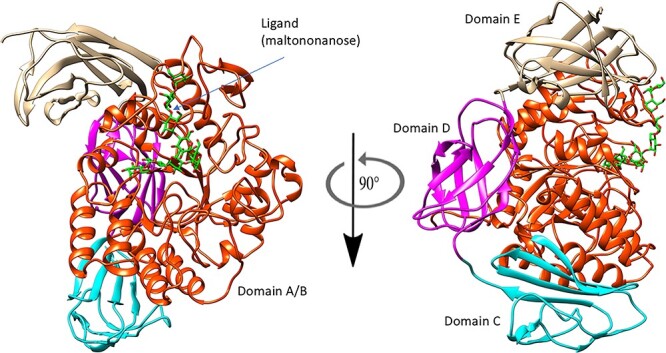
Molecular model of domain A-E in *C*spCGT13. The catalytic A/B domain, in red, shows a ligand (maltononanose) obtained from the atomic coordinates of the crystal structure of the CGTase from *Bacillus circulans* strain 251 (PDB code 1CXK)**.** The D and E domains, typical for CGTases, are shown in purple and light brown, respectively.

**Fig. 2:**
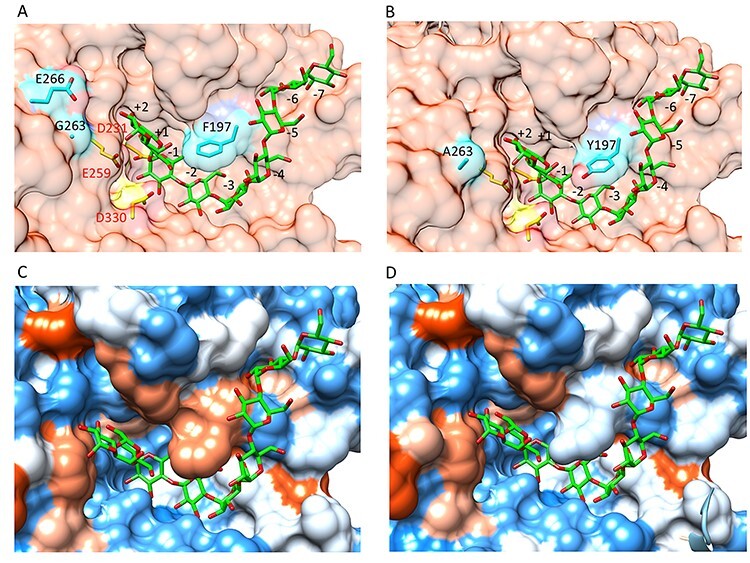
Details of the active site of the modeled *C*spCGT13 structure. (A) Wildtype enzyme showing the predicted catalytic triad (in yellow): Asp 231, Glu 259 and Asp 330, the residues selected for mutations (in cyan): Phe 197, Glu 266 and Gly 263, and the subsites, predicted by superimposing the ligand maltononanose co-crystallized in the structure of the CGTase from *Bacillus circulans* strain 251 (PDB code 1CXK). (B) Mutant F197Y/G263A, which has shown, experimentally, the highest coupling/disproportionation ratio (see also Table IV). (C and D) Hydrophobicity surfaces of the active sites of wildtype (C) and mutant F197Y/G263A (D) are represented according to the Kyte-Doolittle scale (Kyte and Doolittle 1982), from dodger blue for the most hydrophilic, to white, to orange red for the most hydrophobic.

The *C*spCGT13, studied here, contains domains A to E that are conserved for GH13 CGTases, where domains A/B constitute the catalytic domain (Figure 1) (Klein and Schulz 1991; Janecek et al. 1997; Stam et al. 2006; Ara et al. 2015). The domain boundaries of *C*spCGT13 were previously identified (Ara et al. 2015), and in addition to the A/B and C domains typical of most α-amylases in GH13, *C*spCGT13 also contain domain D (lacking well-known function) and the starch-binding domain E, classified under CBM20 (www.cazy.org) typical for the GH13 CGTases (Figure 1). The catalytic A/B-domain consists of a (β/α)8-barrel in which domain B is a loop between β-strand 3 and α-helix 3 of domain A. The catalytic residues (catalytic triad) are located in this domain and are invariably conserved in GH13. In the homology model of *C*spCGT13 the catalytic triad Asp 231, Glu 259 and Asp 330 are positioned at C-terminal extensions of strands β4, β5 and β7 (Figure 2, Panel A). The Asp 231 and Glu259 are the predicted catalytic nucleophile and acid/base.

The substrate binding grove of CGTases contains well preserved substrate-interacting residues, even though some variation in amino acid conservation is found between enzymes from different species (Wind et al. 1998; Kelly et al. 2009). The grove is constructed by nine subsites (shown by a bound maltononaose in Figure 2), numbered from −7 to +2 (Leemhuis et al. 2002; Qi and Zimmermann 2005), with the catalytic triad located in subsite −1. For the CGTase from *T. thermosulfurigenes,* a 10th subsite (+3 subsite) is also mentioned in literature (Leemhuis et al. 2003). D-glucosyl units (e.g. in starch, maltodextrins and cyclodextrins) can bind to these subsites with different affinity depending on the different numbers of interactions in the respective site. By changing the amino acid residues in the subsites, the number of interacting residues will change and alter the binding affinity that will result in altered product specificity in some or all of the reactions catalyzed by CGTases (van der Veen et al. 2001; Leemhuis et al. 2002). In this work, residues at three positions (Phe197, Gly263 and Glu266) in the substrate binding grove were chosen for mutagenesis (Figure 2, Panels A and B). Based on multiple sequences alignment analysis (Figure S1) it is evident that the glycine (Gly) residue at position 263 is highly conserved among other CGTases. On the other hand, at 197 position the residue is either a phenylalanine (Phe, F) or tyrosine (Tyr, Y). A slightly higher residue variation is seen at position 266, and although a glutamate (Glu) is most commonly conserved, there are a few exceptions among the aligned sequences (Figure S1).

For CGTases, the residue corresponding to position 197 has been extensively studied using starch and maltodextrin substrates, and different mutations have been tried. It has been reported that Tyr at this position increases the coupling reactivity (Penninga et al. 1995), although coupling was only studied using methyl-glucoside as the main target acceptor. Changing F197 to Tyr has been hypothesized to allow for interaction with the acceptor substrate in the +1 subsite (Parsiegla et al. 1998). This extra binding to residues in the +1 subsite would help stabilize the acceptor when the binding to the outer +3 subsite is reduced. This would of course help acceptors of varying chain-length, but combined with the increased hydrophobicity, acceptors with short hydrophilic parts would be favored.

Mutation at position 263 has not been studied before in CGTases. The side chain of this residue is oriented toward the acceptor (close to the +2 subsite) (Figure 2, Panels B and D). Thus, the change from Gly to Ala, was hypothesized to increase hydrophobicity in the acceptor subsites. Additionally, energy minimization of the modeled structure including mutation G263A showed that acceptor interaction increased using a long chain acceptor, such as the dodecyl-β-maltoside used in this work. This moves the acceptor toward the area with increased hydrophobicity, which may affect overall binding. The decision to introduce Ala was made since amino acids with larger side chains might introduce steric hindrance in the secondary structure.

Additional change in the hydrophobicity of the acceptor area was created by replacing the Glu at position 266 with Ala. The change at position 266 from Glu to Ala (Figure 2, Panels A and B) was intended for better accommodation of the hydrophobic tail of alkyl glycosides. It has previously been suggested that a mutation at this position can reduce binding of D-glucosyl moiety at a potential +3 subsite, which so far only has been shown to reduce disproportionation activity, while seemingly not effecting the coupling reaction (van der Veen et al. 2001).

### Screening of coupling and disproportionation activity of *C*spCGT13 and its mutants

*C*spCGT13 and the mutants were expressed in *E. coli* and purified by nickel affinity chromatography. In order to understand the effects of the mutations on alkyl glycoside elongation, all the enzymes were screened for coupling and disproportionation activity. Both reactions were studied simultaneously by following the elongation of dodecyl-β-maltoside (β-DDM, C_12_G_2_) using γ-CD as donor (Table I). Since γ-CD contains eight repeating D-glucosyl units, reaction products that are elongated by eight such units (i.e. C_12_G_10_ when using β-DDM as starting material) were considered as primary coupling products. In order to create a simple screening methodology, other reaction products were considered as disproportionation products (following the definition by Ara et al. 2015).

**Table I:** Coupling and disproportionation activity screening of *C*spCGT13 (wildtype) and its mutants

Enzymes	Coupling activity^a^ (U mg^−1^)	Disproportionation^b^ (U mg^−1^)
*C*spCGT13 (wildtype)	94 ± 2	112 ± 5
F197Y	85 ± 2	185 ± 7
G263A	110 ± 1	175 ± 6
E266A	71 ± 2	157 ± 8
F197Y/G263A	150 ± 1	65 ± 5
F197Y/E266A	68 ± 2	63 ± 8
G263A/E266A	115 ± 2	122 ± 5
F197Y/G263A/E266A	142 ± 2	85 ± 5

^a^One unit coupling activity is defined as 1 μmol of primary coupling product (C_12_G_10_) formed per minute.

^b^One unit disproportionation is expressed as 1 μmol of β-DDM consumption per minute not resulting in primary coupling product formation.

Two of the three single mutants (F197Y and E266A) showed a non-desired reduced specific coupling activity in comparison to the wildtype enzyme. Interestingly, F197Y has previously been reported to increase coupling activity (Penninga et al. 1995). The double mutant F197Y/E266A showed a similar decrease in the coupling activity compared to the wildtype. In contrast, the single mutant G263A displayed an increase in coupling activity. Furthermore, this increase in activity was even more pronounced in the two double mutants carrying the G263A mutation and in the triple mutant F197Y/G263A/E266A.

In contrast to predictions based on previous literature data (van der Veen et al. 2001), the measured disproportionation activity was increasing in all the three single mutants, while all double and triple mutants containing F197Y displayed decreased disproportionation activity.

### Kinetic analysis of the coupling reaction

The kinetic parameters of the wildtype and the mutants were determined for the coupling reaction using different concentrations of acceptor substrate (β-DDM) while the concentration of γ-CD was kept constant at 200 mM. Data analysis showed that *C*spCGT13 and its variants followed Michaelis–Menten kinetics. The kinetic data confirmed the high coupling activity of mutant G263A, which displayed a high turnover (Table II).

**Table II:** Kinetic parameters for coupling activity of the wildtype *C*spCGT13 and its mutants. Data presented as means ± standard error from three independent experiments

Enzymes	V_max_ (Umg^−1^)	K_M_^β-DDM^ (mM)	k_cat_ (s^−1^)	k_cat_/K_M_ ^β-DDM^ (s^−1^ mM^−1^)
*C*spCGT13 (wildtype)	109 ± 4.2	15 ± 1.7	137 ± 5.3	**9 ± 0.1**
F197Y	86 ± 7.5	7 ± 0.7	108 ± 9.5	**15 ± 1.3**
G263A	170 ± 6.1	22 ± 3.0	214 ± 10.2	**10 ± 0.3**
E266A	48 ± 4.2	10 ± 2.2	61 ± 5.7	**6 ± 0.2**
F197Y/G263A	117 ± 1.2	13 ± 1.0	147 ± 1.6	**11 ± 0.5**
F197Y/E266A	78 ± 6.7	13 ± 3.0	98 ± 8.4	**8 ± 0.1**
G263A/E266A	129 ± 6.0	20 ± 2.0	163 ± 7.9	**8 ± 0.6**
F197Y/G263A/E266A	153 ± 4.2	15 ± 1.2	193 ± 5.3	**13 ± 0.3**

Interestingly, a significant change in the **K**_**m**_ of acceptor substrate was seen for the single mutations F197Y and G263A with a value of 7 ± 0.7 mM and 22 ± 3.0 mM, which **decreased and increased** the **K**_**m**_ of β-DDM compared to the wildtype enzyme, respectively. The **decreased K**_**m**_ for β-DDM in mutant F197Y was paired with a slight decrease in *k_cat_* compared to the wildtype. A similar trend was observed for the single mutant E266A, while G263A displayed the highest turnover (*k_cat_*) (Table II).

The changes in *k_cat_* and K_m_ were less pronounced for the double and triple mutants, although G263A-containing variants generally displayed a higher *k_cat_* and a similar or increased K_m_ compared to the wildtype (Table II). The coupling reaction by CGTases progresses via ternary complex, in which the **linearized γ-CD donor glycosyl-enzyme and the acceptor (β-DDM) bind simultaneously**. Based on the kinetic parameters for the acceptor molecule it is evident that mutations at the central position and both +2 and +3 subsites affected the coupling activity.

**Fig. 3:**
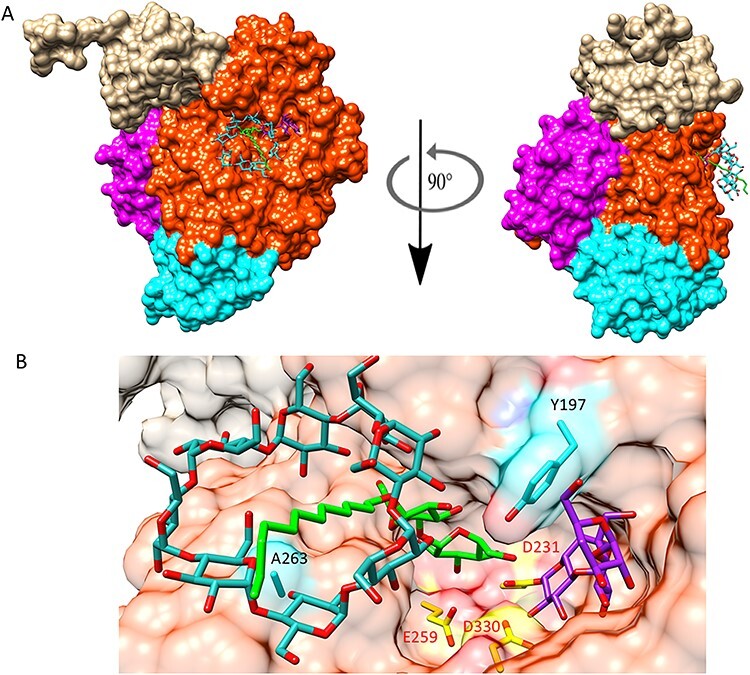
Molecular model of the complex of *C*spCGT13/γ-cyclodextrin/dodecylmaltoside/covalent intermediate maltotrioside. (A) Overall view of the complex. Domains A and B are shown in red, domain C in cyan, D in purple and E in light brown. The γ-cyclodextrin is represented with blue sticks, carbon atoms in cyan and oxygen in red and the dodecyl-maltoside carbons are in green with oxygens in red. (B) Close-up view of the interactions of ligands with the double mutant F197Y/G263A. The Maltotrioside covalently bonded to the aspartate 231 is represented with carbons in dark purple and oxygens in red. Catalytic triad Asp 231, Glu 259 and Asp 330 is colored in yellow. In this complex the alkyl chain of the dodecyl-maltoside is shown to be captured by the γ-cyclodextrin ring.

In previous work, the alkyl chain in alkyl glycosides of low water solubility was shown to be complexed with non-reacted CDs in the reaction mixture (Börner et al. 2014) (the alkyl chain captured inside the CD ring), drastically effecting their conversion by different glycoside hydrolases (Rather et al. 2015). The CD-DDM interaction simplifies solubilization but could also result in surface interactions between the CD and the enzyme (Figure 3). Modeling data obtained after docking of such a complex (a β-DDM placed inside a γ-CD ring) showed that the G263A substitution may result in an interaction with the β-DDM-CD complex (Figure 3, Panel B). The kinetic data (a higher K_m_), however, showed that in fact this must have led to an overall loss of affinity in the interactions between the β-DDM acceptor and the residues in the acceptor sub sites. The increased turnover may then be consequence of a faster release of the obtained product.

The modeling data also showed that substitution of Phe at position 197 for Tyr can potentially establish hydrogen bonding with the D-glucosyl unit of the β-DDM (Figure 3, Panel B) and hence help in the stabilization of the acceptor molecule. The lower K_m_ may reflect the hydrogen bonding, but this also resulted in a decrease in turnover.

### The influence of disproportionation and hydrolysis

Parallel with the coupling process, two major side reactions take place: disproportionation, where the elongated primary coupling product act as a donor, and hydrolysis (of either the γ-CD resulting in linear oligosaccharides, or of produced coupling products). Consequently, the actual product profile is quite complex and difficult to disentangle in mechanistic terms (Figure S2). Within the scope of the current work, it was not possible to identify alkyl glycosides products formed *solely* by disproportionation, and the role of hydrolysis needs to be kept in mind. The apparent increase in disproportionation activity observed in the single mutants and in the G263A/E266A-double mutant (Table I) could thus potentially be the result of combined hydrolysis and disproportionation reactions.

The specific hydrolysing activity of *C*spCGT13 and the mutants were determined by using 1% wheat starch (Table III). No significant decrease in hydrolysis was observed, but a small increase in hydrolysis activity could be seen in the F197Y and G263A variants. This shows that the mutations in acceptor subsites would likely only have a minor influence on hydrolysis. However, as mutants involving G263A have an **increased K**_**m**_ for the β-DDM acceptor, competition with CD and oligosaccharide residues may promote disproportionation of oligosaccharide products.

**Table III:** Hydrolytic activity of *C*sp*CGT13* and its mutants. The hydrolysis was done using 1% wheat starch

Enzymes	Hydrolytic activity (U mg^−1^)
*C*spCG13	12.7 ± 0.0
G263A	15.4 ± 0.6
E266A	12.5 ± 0.6
F197Y	15.1 ± 0.7
G263A/E266A	13.5 ± 0.2
F197Y/G263A	15.3 ± 0.6
F197Y/E266A	14.5 ± 0.9
F197Y/G263A/E266A	13.8 ± 0.4

As our focus was to increase the coupling in relation to all other products in the mixture, the total reaction rate and the ratio of coupling over total reaction rate was determined for the double and triple mutants together with the wildtype *C*spCGT13 (Table IV). Still, even if the differences were less apparent, the highest ratio of coupling over total reaction rate was for the mutant F197Y/G263A (Table IV), in accordance with the results in Table I. This may be due to a beneficial combination of the affinity increasing F197Y mutation (**decreased K**_**m**_) with the affinity decreasing G263A (**increased K**_**m**_).

**Table IV:** Total reaction and ratio of coupling for *Csp*CG13 (wildtype) and its mutants. The data in this table are derived from Table I

Enzymes	Total activity^a^ (U mg^−1^)	Coupling^b^/total activity
*C*spCG13	206.6± 6.3	0.47 ± 0.01
F197Y/G263A	215.1± 4.0	0.70 ± 0.00
F197Y/E266A	131.5± 5.6	0.53 ± 0.01
G263A/E266A	236.6 ± 6.5	0.49 ± 0.00
F197Y/G263A/E266A	226.8± 8.0	0.63 ± 0.01

^a^Total activity refers to the sum of coupling and disproportionation activity.

### Effects of the mutations on the production of primary coupling product

**Fig. 4:**
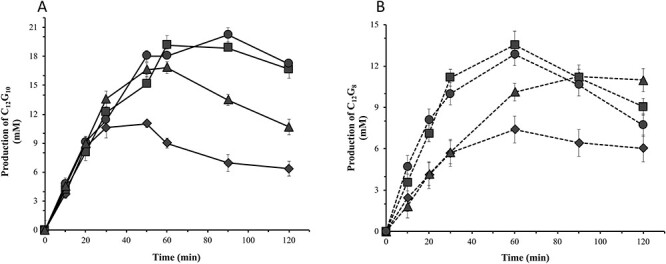
Time course of production of primary coupling products by *C*spCGT13 and its mutants. In both cases the reaction was initiated by adding 5 μg/ml of enzymes. The wildtype *C*spCGT13 is represented by filled diamonds and the mutants F197Y/G263A by filled squares, G263A/E266A by filled triangle and F197Y/G263A/E266A by filled circles. (A) production of C_12_G_10_ using 200 mM gamma cyclodextrin (γ-CD) as donor and 50 mM dodecyl-β-maltoside (β-DDM) as acceptor. (B) production of C_12_G_8_ using 200 mM alpha cyclodextrin (α-CD) as donor and 50 mM dodecyl-β-maltoside (β-DDM) as acceptor.

The primary coupling product (C_12_G_10_) obtained in reactions with wildtype *C*spCGT13 and selected mutants using β-DDM as acceptor and γ-CD as donor was analyzed in a time course study (Figure 4A). The study of the mutants F197Y/G263A/E266A and F197Y/G263A showed the maximum concentration of C_12_G_10_ to appear later than for wildtype, with no or little reduction of the product upon extended incubation. On the other hand, wildtype *C*spCGT13 and G263A/E266A had a lower maximum concentration and more significant reduction of the product with extended incubation time (Figure 4A), indicating conversion to different secondary reaction products. Since *C*spCGT13 also shows substantial coupling activity with α-CD (Ara et al. 2015; Rather et al. 2015), this donor was evaluated, using the G263A/E266A, F197Y/G263A and F197Y/G263A/E266A mutated enzymes, respectively, as catalyst (Figure 4B). The reactions were run using β-DDM as acceptor and α-CD as donor, expecting C_12_G_8_ as the primary coupling product. Again, the F197Y/G263A and F197Y/G263A/E266A mutants produced the highest concentration of C_12_G_8_ (14–13 mM), while the wildtype *C*spCGT13 reached a maximum of 7 mM (Figure 4B.). The consumption of the primary coupling product, C_12_G_8_, was however significant in comparison to C_12_G_10_, a clear indication of disproportionation reaction using C_12_G_8_ as donor and acceptor. Overall, it is also evident that the wildtype *C*spCGT13 and the variants prefer γ-CD as donor for the elongation of alkyl glycosides.

### Dodecyl-β-glucoside as acceptor substrate

**Fig. 5:**
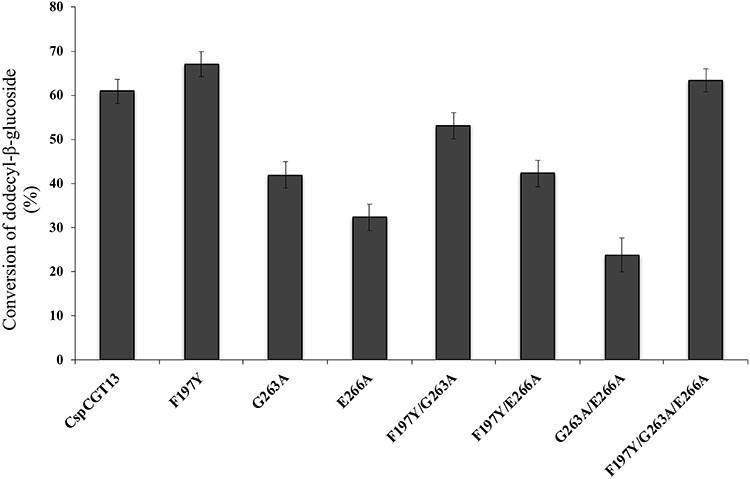
Conversion of dodecyl-β-glucoside (DDG) into dodecyl-β-glycosides using γ-cyclodextrin as donor. The reaction was initiated by adding 5 μg/mL of the wildtype (*C*spCGT13) and its mutants and continued for 2 h. The concentration of the donor (γ-CD) and acceptor (DDG) was 200 and 50 mM, respectively.

Dodecyl-β-glucoside (β-DDG) is more hydrophobic than its corresponding maltoside and has poor solubility in water. Since the substitution to the more hydrophobic residue at position 263 increased the amount of elongated coupling product, additional experiments were made to evaluate the possibility to use β-DDG (C_12_G_1_) as acceptor in corresponding reactions. Reaction conditions were identical to the screening trials using β-DDM, except that β-DDG was used as the acceptor. The results showed a 7% increase in the conversion of DDG into β-dodecyl glycosides using γ-CD as donor and the F197Y mutant (compared to the wildtype enzyme) followed by a 2% increase by the triple mutant (Figure 5). The changes in the +2 subsite (position 263) were less beneficial using this acceptor. This indicates that the interaction with the acceptor with a single D-glucosyl moiety is significantly more affected by the single substitution at position 197 (Figure 3, Panel B), coinciding with the earlier hypothesis based on use of methyl glucosides, indicating the importance of the length of the hydrophilic head of the acceptor for the coupling efficiency.

## Conclusions

In conclusion, by using a rational mutagenesis approach we were able to select two mutants F197Y/G263E and F197Y/G263E/E266A with improved coupling activity and decreased disproportionating activity using an alkyl glycoside acceptor with a long alkyl chain, and a maltoside head-group. The mutations at +2 and the potential +3 subsites can affect the acceptor substrate binding affinity but had little influence on starch hydrolysis activity. It was evident that residue 263 plays a role in tuning the affinity of the alkyl chain of the dodecyl maltoside, resulting in increased turnover in the coupling reaction. On the other hand, F197Y is important for making interactions with the D-glucosyl residues of the alkyl glycosides, resulting in increased coupling using single D-glucosyl moiety acceptor. The mutation E266A at the potential +3 subsite did not significantly influence coupling or starch hydrolysis activity, but could have an influence when combined with the F197Y substitution.

## Materials and methods

### Materials

α-Cyclodextrin (α-CD) and γ-Cyclodextrin (γ-CD) were purchased from Wacker Chemie AG, Munich, Germany. Dodecyl-β-maltoside (β-DDM) and Dodecyl-β-glucoside were sourced from Anatrace Inc., Maumee, OH, and wheat starch from Sigma-Aldrich, St. Louis, MO. Synthesis of dodecyl-β-maltooctaoside (C_12_G_8_) is described by Rather et al. (2015). All other chemical and solvents were HPLC grade and purchased from VWR International, Stockholm, Sweden.

### Site-directed mutagenesis

The mutation process was done in three steps. In the first step single amino acid changes were introduced at three different positions, Gly was changed to Ala at position 263 (G263A), Glu to Ala at position 266 (E266A) and Phe to Tyr at position 197 (F197Y). In the second step combinations of two mutations were made, creating three double mutants. Finally, one triple mutant was created, using a combination of all three mutations. This three-step setup gives the ability to reuse the primers for each step, except when G263A is introduced to a mutant with E266A present or vice versa. In this case, due to the fact that both mutated residues are separated only by a few amino acids, a single primer was used for both mutations (Table V). The *Csp*CGT13 gene was cloned into expression vector pJOE3075 (Ara et al. 2015). The mutants were generated through synthetizing the plasmid by polymerase chain reaction (PCR), using a methylated template and the mutagenic primers listed under Table V. Phusion High-Fidelity DNA Polymerase (Thermo Scientific, MA) was used for amplification with recommended conditions by the manufacturer. Afterwards, the PCR reaction mixes were treated with DpnI endonuclease, at 37°C per 1 h, in order to digest the wildtype template. The PCR products were transformed into XL1-Blue supercompetent cells (Agilent, Santa Clara, CA) for the proliferation of the plasmids. Subsequently, the plasmids were purified using the E.Z.N.A plasmid mini kit I (Omega bio-tek, Norcross, GA) and sequenced for the verification of the mutations.

**Table V:** List of primers used for the generating mutants of *C*spCGT13

Mutations	Primers
F197Y	5’-GAGGATGGAATTTACAAAAATTTATATGATTTAGCAGACCTAAAC-3’
	5’-GTTTAGGTCTGCTAAATCATATAAATTTTTGTAAATTCCATCCTC-3’
G263A	5’-GGTGAATGGTTCTTAGCTGTTAATGAAGTAGATCAAAACAAC-3’
	5’-GTTGTTTTGATCTACTTCATTAACAGCTAAGAACCATTCACC-3’
E266A	5’-GGTGAATGGTTCTTAGGTGTTAATGCAGTAGATCAAAACAAC-3’
	5’-GTTGTTTTGATCTACTGCATTAACACCTAAGAACCATTCACC-3’
G263A/E266A	5’-GGTGAATGGTTCTTAGCTGTTAATGCAGTAGATCAAAACAAC-3’
	5’-GTTGTTTTGATCTACTGCATTAACAGCTAAGAACCATTCACC-3’

### Expression and purification

The wildtype *Csp*CGT13 from *Carboxydocella* sp. and mutant proteins were expressed in *E. coli* BL21 (DE3) strain (Novagen, Merck group, Darmstadt, Germany). The transformations of the plasmids were performed by thermal shock at 42°C, according to the manufacturers’ instruction (Novagen). Cells harboring the plasmids were grown in 1 L baffled shake flasks with 100 mL Luria Bertani medium supplemented with 100 μg/mL ampicillin at 37°C. The protein expression was induced at OD _600nm_ = 0.5 with 0.2% L-rhamnose. After 15 h production phase the cultures were harvested by centrifugation. To lyse the cells 5 mL Bugbuster (Novagen) lysis solution was added per gram cell pellets (wet weight), re-suspended and incubated at room temperature for 30 min. Afterwards, cell extracts were centrifuged at 13,000 rpm × 20 min and the supernatant was collected for purification.

The protein purification step was done by immobilized metal ion affinity chromatography (IMAC) using an ÄKTA Start™ system with a HisTrap™ FF crude column (GE Healthcare, Uppsala, Sweden) as described previously (Ara et al. 2015). Purity of the proteins was estimated by SDS-PAGE and their respective concentration was measured by Bradford reagents using bovine serum albumin as standard.

### Molecular modeling

A homology model of *C*spCGT13 was built using the YASARA software (Krieger et al. 2002; Krieger and Vriend 2014) with default settings (Supplementary Table I). All possible templates were identified by running 25 PSI-BLAST iterations to extract a position specific scoring matrix (PSSM) from UniRef90 and searching the PDB for a match. The best matching template structures (Table VI) were selected based on the BLAST alignment score. **YASARA homology modeling generates five alignments for each template and builds a model for each alignment. A hybrid model is then generated by combining the best parts of the models**. Unrestrained refinement of the full model was performed by energy minimization with explicit solvent molecules of water. The models from the respective template were ranked based on the *z*-score, which measures the difference between the model and the average high-resolution X-ray structure, based on standard deviation. A negative z-score indicates low quality with respect to the high-resolution X-ray structure. The overall *z*-score is a weighted average of individual *z*-scores capturing correctness of backbone, side-chain dihedrals and packing interactions. Finally, the best parts of the respective model were combined, resulting in a hybrid model with a better *z*-score (Supplementary Table II). The hybrid model was refined by short time (500 ps) molecular dynamic simulations in water, following the protocol described by Krieger et al. (2004).

**Table VI:** Templates used for building the hybrid model of *C*spCGT13

Template	PDB-entry	Source	Coverage (%)	Align score	Total score
1	3BMW-A	*Thermoanerobacterium thermosulfurigenes* Em1	98	2798.0	1302.20
2	1CXL-A	*Bacillus circulans* Strain 251	98	2613.0	1161.48
3	1PJ9-A	*Bacillus circulans* Strain 251 Loop mutant183–195	98	2609.0	1159.71
4	1V3M-A	*Bacillus* sp. strain 1011 mutant F283Y	98	2540.0	1158.94
5	1OT1-A	*Bacillus circulans* Strain 251 mutant D135A	98	2608.0	1156.70

### Coupling and disproportionation activity

The coupling and disproportionation activity were determined using 50 mM of β-DDM or β-DDG as acceptor substrate and 200 mM of γ-CD or α-CD as donor. The reactions were carried out at 1 mL scale in 4.5 mL capped glass vials using a thermo-shaker (Hettich Benelux Heating Thermo Shaker MHR 13, Geldermalsen, Netherlands) set at 60°C and shaking at 600 rpm. The buffer used for the reaction was 10 mM sodium-citrate buffer (pH 6) unless otherwise stated and with addition of 2 mM CaCl_2_. The initiation of the reaction was done using 5–6 μg/mL of enzymes and 50 μL aliquots of reaction mixture were withdrawn at different time points. The aliquots were then mixed with 950 μL of dimethyl sulfoxide (DMSO) to stop the reaction after 20 min. All the samples were analyzed by high performance liquid chromatography with a charged aerosol detector (HPLC-CAD). During coupling reaction, C_12_G_8_ and C_12_G_10_ formed as primary coupling products when using β-DDM as acceptor and α and γ cyclodextrins as donor, respectively. Both coupling and disproportion activities were determined using initial reaction rates. One unit of coupling activity is defined as 1 μmol of primary coupling product formed per minute. To allow for comparison of coupling and disproportionation, disproportionation activity was identified as follows: one unit corresponding to 1 μmol of β-DDM consumption per minute, not resulting in formation of primary coupling product, in accordance with the definition used in Ara et al. (2015). The concentrations of β-DDM and dodecyl-β-D-maltooctaoside (β-DDMO, C_12_G_8_) were determined by using six-point external standard curves. The concentration of C_12_G_10_ was calculated by using the dodecyl-β-D-maltooctaoside standard curve.

### Kinetic analysis of coupling activity

Kinetic analysis was carried out for coupling activity at reaction condition mention above. For this analysis β-DDM was used as acceptor and γ-CD as donor. The concentrations of β-DDM were varied from 10 to 50 mM, while the concentration of donor γ-CD was kept constant at 200 mM. The kinetic parameters K_M_ and kcat were determined by using KinTek Explorer.

### HPLC-CAD analysis

Alkyl glycosides were analyzed using a Dionex HPLC system (Thermo Scientific Dionex UltiMate®3000) provided with a C18-RP column (Acclaim ™ Vanquish™ C18, 2.2 μm 120 Å, 2.1×150 mm, Thermo Scientific) and connected to a CAD operating at evaporation temperature 35°C with an operating gas pressure of 60.7 psi. Elution was carried out using a gradient of acetonitrile (B) and 0.1% (v/v) acetic acid in milli Q water (A) at a flow rate of 0.4 mL/min and an injection volume of 5 μL. The gradient of eluent (B) started at 25% and kept constant for 5 min followed by an increase to 80% in 20 min. It was held at 80% for another 5 min and then returned to 25% in 5 min and was kept steady for 5 min before the start of the next run.

### Hydrolysis

Hydrolysis activity was determined by the 3,5-dinitrosalicylic acid (DNS) reagent according to the method described in Ara et al. (2015), using 1% (w/v) wheat starch (Sigma). 1 unit of hydrolytic activity is defined as 1 μmol of maltose formed per minute and all the reactions were run in triplicates.

## Supplementary Material

Supplementary_Information_cwaa109
