# Supplementary material for: Engineering CGTase to improve synthesis of alkyl glycosides
Source: Glycobiology. 2020 Dec 2;31(5):603–12. doi: 10.1093/glycob/cwaa109 (PMC8176775; doi:10.1093/glycob/cwaa109)
Supplement: Supplementary_Information_cwaa109 [file supplementary_information_cwaa109.pdf]

Supplementary information

Engineering CGTase to improve the synthesis of alkyl glycosides

Kazi Zubaida Gulshan Ara[1], **Javier A. Linares-Pastén**[1], **Jonas Jönsson**[1], **Maria Viloria-Cols**[1,2], **Stefan Ulvenlund**[2], **Patrick Adlercreutz**[1], **Eva Nordberg Karlsson**[1]

[1]Biotechnology, Dept. of Chemistry, Lund University, P.O. Box 124, Lund, Sweden

[2]Enza Biotech AB, Scheelevägen 22, Lund, Sweden

Corresponding author:

Eva Nordberg Karlsson

Email: eva.nordberg_karlsson@biotek.lu.se

Telephone: +46 46 222 4626

Kazi Zubaida Gulshan Ara

Email: zubaida.gulshan_kazi@biotek.lu.se

Keywords: Acceptor subsites; Cyclodextrin glycosyltransferase; Coupling reaction; Dodecyl-β-maltoside; γ-Cyclodextrin.

Supplementary figure legends

Supplementary Figure 1: Multiple sequence alignment of CGTases from different organisms showing conservation of residues at position 197, 263 and 266. Residue numbers are according to *C*spCGT13 from *Carboxydocella* sp..

Supplementary Figure 2: Product profiles of the *C*spCGT13 and its variants during elongation of dodecyl-β-maltoside. The profile displays relative peak area (%)of products formed after 30 min of reaction using dodecyl- β-maltoside as acceptor and gamma CD as donor. The reaction was initiated by using 5-6 µg of enzymes. $C_{12}G_1$: C_{12} represents the length of alkyl chain which is dodecyl whereas G_{1-17} represents the degree of polymerrization of the D-glucosyl unit in carbohydrate moeity.

Supplementary Figure 1

```
                                                                                                                197
Carboxydocella sp. CspCGT13(AIL25792.1)                     QSDASYAENGKLYDNGVLLASYSNDPNGLFHHNGGTDFSTIEDGIYKNLFDLADLNHQNN 207
Bacillus circulans 251(P43379)                              SDQPSFAENGRLYDNGTLLGGYTNDTQNLFHHNGGTDFSTTENGIYKNLYDLADLNHNNS 232
Bacillus sp.8SB (P30920)                                    ETDTSFAENGRLYDNGTLVGGYTNDTNGYFHHNGGSDFSSLENGIYKNLYDLADFNHNNA 239
Paenibacillus macerans (P31835)                             STDPSFAENGALYNNGTLLGKYSNDTAGLFHHNGGTDFSTTESGIYKNLYDLADINQNNN 232
Thermoanaerobacter sp. ATCC 53627(CAJ84380.1)               ETDPTYGENGRLYDNGVLLGGYTNDTNGYFHHYGGTNFSSYEDGIYRNLFDLADLDQQNS 206
Thermoanaerobacterium thermosulfurigenes EM1(P26827)        ETDPTYAENGRLYDNGTLLGGYTNDTNGYFHHYGGTDFSSYEDGIYRNLFDLADLNQQNS 233
Paenibacillus macerans IFO 3490(P04830)                     RDNPGFAENGGMYDNGSLLGAYSNDTAGLFHHNGGTDFSTIEDGIYKNLYDLADINHNNN 232
Bacillus clarkia (B9A1J6)                                   ------IEDGALYDNGTLLGHYSTDANNYFYNYGGSDFSDYENSIYRNLYDLASLNQQHS 224

                                                                                                                       263 266
Carboxydocella sp. CspCGT13(AIL25792.1)                     TIDSYMKAAIKKWLDLGIDGIRMDAVKHMPFGWQKNFMDSIYS-YKPVFTFGEWFLGVNE 266
Bacillus circulans 251(P43379)                              TVDVYLKDAIKMWLDLGIDGIRMDAVKHMPFGWQKSFMAAVNN-YKPVFTFGEWFLGVNE 291
Bacillus sp.8SB (P30920)                                    TIDKYFKDAIKLWLDMGVDGIRVDAVKHMPLGWQKSWMSSIYA-HKPVFTFGEWFLGSAA 298
Paenibacillus macerans (P31835)                             TIDSYLKESIQLWLNLGVDGIRFDAVKHMPQGWQKSYVSSIYSSANPVFTFGEWFLGPDE 292
Thermoanaerobacter sp. ATCC 53627(CAJ84380.1)               TIDSYLKAAIKLWLDMGIDGIRMDAVKHMAFGWQKNFMDSILS-YRPVFTFGEWYLGTNE 265
Thermoanaerobacterium thermosulfurigenes EM1(P26827)        TIDSYLKSAIKVWLDMGIDGIRLDAVKHMPFGWQKNFMDSILS-YRPVFTFGEWFLGTNE 292
Paenibacillus macerans IFO 3490(P04830)                     AMDAYFKSAIDLWLGMGVDGIRFDAVKHMPFGWQKSFVSSIYGGDHPVFTFGEWYLGADQ 292
Bacillus clarkia (B9A1J6)                                   FIDKYLKESIQLWLDTGIDGIRVDAVAHMPLGWQKAFISSVYD-YNPVFTFGEWFTGAQG 283
```

Supplementary Figure 2

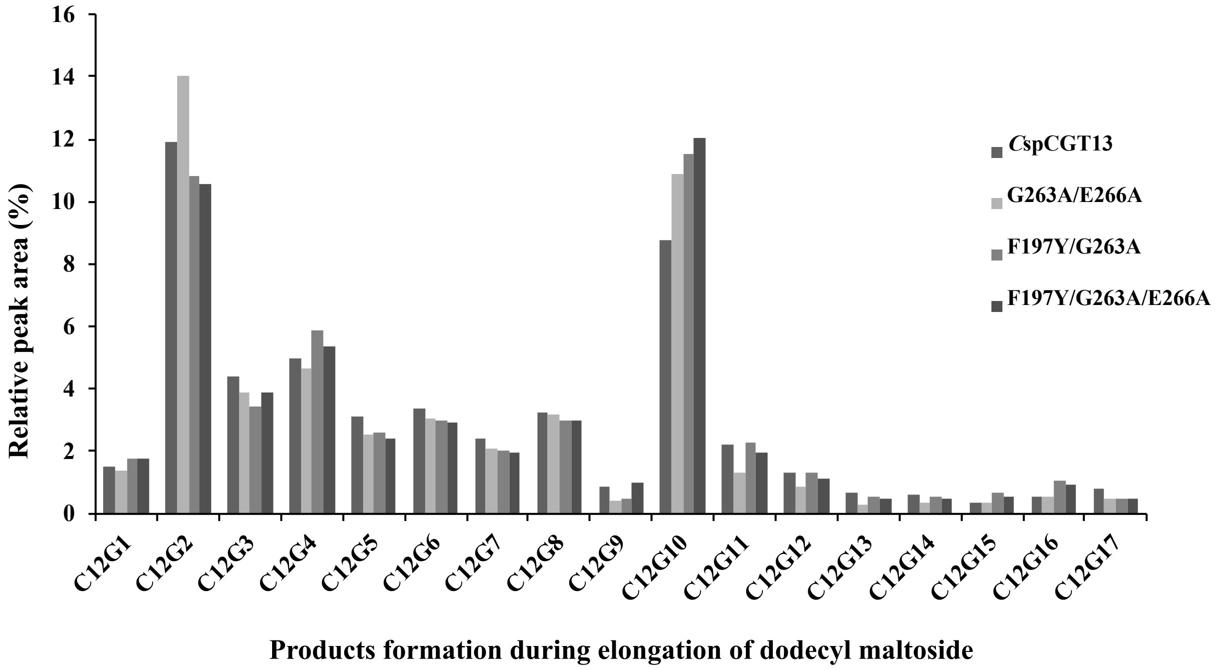

Supplementary Table I. Homology modelling parameters for *C*spCGT13

**Parameter**	**Description**
Modeling speed	Slow
PSI-BLAST iterations in template search	3
Maximum allowed (PSI-)BLAST E-value to consider template (EValue Max)	0.5
Maximum number of templates to be used	5
Maximum number of templates with same sequence	1
Maximum oligomerization state	4
Maximum number of alignment variations per template	5
Maximum number of conformations tried per loop	50
Maximum number of residues added to the termini	10

Supplementary Table II: Validation of the *C*spCGT13 model

**Check type**	**Quality Z-score**	**Comment**
Dihedrals	1.030	Optimal
Packing 1D	0.179	Optimal
Packing 3D	-1.733	Satisfactory
Overall	-0.587	Good
